# Editorial

**Published:** 1873-04

**Authors:** 


### EDITORIAL.

### A Diseased Mouth A Source of General Disease.

It is an established medical observation that diseases of the mouth and associate parts often arouse severe and protracted morbid action in other parts of the body. That organs which have important physiological sympathies with the whole nutritive, and a large portion of the nervous system, should, through this close relationship, and when very much diseased, excite morbid action in other parts, is what we might expect. This is the case in many instances of only moderate disease, but it must be especially so in cases of extensive breaking up of the dental arch, where not only are the gums tumid with putrid blood and pus, the sockets and periostea involved, but the maxillary bones and the sinuses are encroached upon by disease, and the whole track of the fifth pair of nerves is the seat of severe functional derangement. The powerful morbic influence which such a state of things must exert on the general constitution is not likely to be fully appreciated until the subject is carefully considered.

The journals occasionally, and the practitioner's note-book constantly, contain cases in which violent neuralgic affections of the face, great nervous depression, loss of digestion, and general prostration, one or the other, or all of these conditions, have attended diseased states of the teeth and contiguous parts.

The neuralgia is accounted for by the existence of numerous lesions in the filaments of the trifacial nerve; and in this alone we have a source of great constitutional disturbance through the ineffectual efforts of nature to repair the injuries under which she suffers. A drain upon the system, so heavy and so perpetual, must impair the nervous supplies which go to other organs. And here we have the foundation of gastric derangements, biliary disorders, and constipation, which in their turn must undermine and break up the vigor of all the functions, and the healthy activity of the whole organism.

And this state of things will be aggravated by morbid compounds of vitiated secretions, which, along with drippings of blood which has stagnated in the gums, will be carried into the stomach to impair its digestive fluids, and along with unchewed aliments, derange the action of the organ.

The little attention given to these general morbific effects, arising from diseased conditions of the mouth and asssociate parts, may be due to a disposition on the part of practitioners to look for more profound causes of disease. The note book of the dental practitioner is replete with cases that might be cited in illustration.

### Professional Literature.

In looking over the past and present of dentistry, it is easy to see why we still lack able works on several important special subjects: there has not been time for their production.

It is only during recent years that we have become awakened to the breadth and complexity of the problems that present themselves to the dentist for solution; it is only recently that we have realized the difficulties that must be encountered in solving these problems.

Inquiries relating to the morphology, physiology, and pathology of dentine, its molecular defects, its modifications in disease, and under different remedial influences; the questions of form, development, and life-endowment, that arise from the study of both dentine and enamel; many chemico-vital problems, involving study in dietetics, and the wonderful influences that are impressed upon the delicate dental germs during gestation, are some of the unworked, or but partially wrought territories of dental investigation. In these and other unnamed subjects of inquiry, are mines of rich materials for the man of science; and many an unclaimed, though fertile spot, on which the accurate observer and close thinker may plant himself, with test-tube and microscope, to make for himself a secure place in the history of this new specialty of medicine.

Many subjects have yet to be made clear: the influence of diet on the teeth; the facts of histology; the laws of restoring lost features; the assimilation of the phosphates, and a multitude of topics in dental medicine.

We have as yet no work on diagnosis, none adequate on dental therapeutics, and a number of special departments in dental pathology already await their accurate observer and recorder.

### A New Dental Journal and a Dental College in Spain.

Just before going to press we have received a new dental journal form Madrid. It is edited by Dr. Cayetano Trivano, a member of the Academy of Surgeons, and author of a work on Dental Surgery, and is called the *Revisita Odontalgica* It is well printed, and contains the usual articles and discussions on anæsthetics, extraction, dental charlatanism, etc., such as fill our own journals.

A Spanish dental college has been established, or is being established by a society of surgeons, with Dr. Louis Portella at its head, who is also President of the Academy of Medicine and Surgery in Madrid. This dental Faculty is to be instituted somewhat on the American plan, but will be more regulated by the government.

### Reorganization of the Cincinnati Dental Association.

The following is a synopsis of the proceedings at a meeting of dentists, held March 24th, 1873, at Dr. J. Taft's office, for the purpose of reorganizing the Cincinnati Dental Association. There were present, Drs. Berry, Goe, Hawxhurst, Meredith, Roudebush, H. A. Smith, H. R. Smith, Taft, Taylor and Wheeler.

Dr. Roudebush was appointed President, pro tem., and Dr. Meredith, Secretary, pro tem. Dr. Taft read the old constitution of Jan. 6th, 1844. Meetings were held regularly under this for one year, then discontinued.

He read another constitution of 1848, and stated that it was probably the old one revived. Meetings were held under it for several years.

Dr. Smith read minutes of two meetings held in 1865 and 1867.

Dr. Taft moved that the last constitution should be the one adopted for present government. Carried.

It was moved and carried that old members practicing in the city should still be regarded as members.

Dr. Hawxhurst, Goe, and Meredith were nominated for membership, and elected.

The Association then proceeded to the election of permanent officers, for six months from date. The result was as follows:

Drs. H. R. Smith, President; B. D. Wheeler, Vice President; D. C. Hawxhurst, Secretary; H. A Smith, Corresponding Secretary; A. Berry, Treasurer.

Drs. F. Hunter, L. P. Meredith, and H. A. Smith, were elected an Executive Committee.

Drs. Meredith and Hawxhurst, were selected to read papers at the meetings of March 31, and four weeks from that date, respectfully.

L. P. Meredeth, Secretary.

### The Vulcanite Question.

Frequent inquiries are made in reference to the present status of the claims of the Dental Vulcanite Company. Many have an impression, since the recent action of the Supreme Court at Washington, that the Cummings patent is void.

The recent action of the Supreme Court was upon the affidavits of S. S. White and others, bringing out facts that had not before been presented fully, for the purpose of considering the dismissal of the appeal.

We give below the motion for dismissal, also the opinion of the Court reversing the former judgment.

This opinion is based upon the evidence of collusion, between the Dental Vulcanite Company and those conducting the defense, which is most damaging to both parties, and presents Mr. Bacon & Co. in a worse light, if possible, than before.

In all this, however, the validity of the Cummings patent is not invalidated, nor materially affected, except so far as such conduct on the part of the Dental Vulcanite Company is a remarkable admission of the inherent weakness of that patent. We do not believe there is a man living, who is at all familiar with the history of the matter, who does not regard it as a fraud throughout. And Mr. Bacon is afraid to trust the result of a fair trial of the cause upon its merits, and the testimony that may even now be had. We take the following from advance sheets of the *Dental Cosmos:*

---

### UNITED STATES SUPREME COURT.

No. 133. December Term, 1871.

BENONI E. GARDNER, *Appellant,*
*vs.*
THE GOODYEAR DENTAL VULCANITE COMPANY AND JOSIAH BACON.
} *Motion to dismiss appeal.*

And now this twenty-ninth day of October, A. D. 1872, come before the court, Jeremiah S. Black and Henry Baldwin, Jr., on behalf of Samuel S. White, of Philadelphia, Pennsylvania, and of many others, dentists, all citizens of the United States, and move the court to dismiss this appeal.

And in support of this motion they produce in court the affidavits of Benoni E. Gardner, this appellant, John B. Newbrough, Henry E. Fickett, Henry Coy, and Samuel S. White; and thereupon show the following facts:

1st. That there was no dispute between the plantiffs and the defendant in the court below at the time the final decree was there made.

2d. That there was no dispute between the appellant and the appellees in this court at the time this appeal was taken, nor when it was argued.

3d. That the decree for damages and costs against the defendant in the court below was not, and is not intended to be inforced, but that this appellant was and is protected against the same by agreements made in that behalf by the appellees before said decree was taken.

4th. That the injunction granted by the court below in its final decree was not and is not intended to be enforced, but that the appellant was protected against the same by a license granted in that behalf by the appellees before said decree was taken.

5th. That the appellant is not even liable to costs in this court, but that the same have been or are to be paid by the appellees.

6th. That the appellant here has no interest whatever in the questions involved in this appeal, nor in their determination; but that the appellees herein are attempting to procure the opinion of this court upon questions of law, in the decision of which their interest is opposed to that of other persons who are not parties to this suit, who had no knowledge of it while it was pending in the Circuit Court, and no opportunity of being heard in defense of their rights.

7th. That this cause was argued in the court below, and in this court, without there being any interest adverse to that of the appellees represented therein, and without there being even a colorable dispute, to obtain an opinion of this court, which the appellees desire to have in their own interest, and for their own purposes, when there is no real and substantial controversy between them and the appellant, and no conflict between their interests; all the interest involved herein pertaining to the appellees, and that interest adverse to, and in conflict with the interest of third parties, whose rights would be seriously impaired if the question of law herein are decided in the manner in which these appellees have sought, and are seeking to have them adjudicated upon this appeal.

8th. That the Goodyear Dental Vulcanite Company became and was in the court below, and has been, and is in this court, the sole party in interest in this cause, and as such *dominus litis* on both sides thereof.

9th. That while the cause was pending in the court below the appellees became the legal and equitable owners of all and any opposing interest at any time involved therein; employed and paid counsel on both sides, and made up a record therein to suit themselves; in order to obtain an opinion of this court that should conclude the rights and interests of persons not parties to the pretended controversy.

Upon the facts above stated, the counsel named, on behalf of their clients aforesaid, move the court to strike out the judgment or decree heretofore made in the said cause, and to quash the appeal taken therein, direct the Circuit Court to dismiss the plaintiff's bill, and take such measures in the premises as justice and right may seem to require.

J. S. BLACK,
HENRY BALDWIN, JR.

*Indorsed*: Supreme Court U. S., 1871, Dec. Term, No. 133. Gardner, app't *vs*. Dental Vulcanite Company. Motion to set aside decree and dismiss appeal. Filed 29th October, 1872.

True copy.

[SEAL.] TEST.: D. W. MIDDLETON,
*Clerk Supreme Court U. S.*

[This decision was rendered Monday, March 3. 1873.]

SUPREME COURT OF THE UNITED STATES.

No. 133 December Term, 1871.

| | |
|---|---|
| BENONI E. GARDNER, *Appellant*, vs. THE GOODYEAR DENTAL VULCANITE COMPANY, *et al.* | *Appeal from the Circuit Court of the United States for the district of Rhode Island.* |

*On Motion.*

Mr. Chief Justice Chase delivered the opinion of the court.

The original suit in equity was brought by the Goodyear Dental Vulcanite Company against Gardner to enjoin him from the use of certain patented subjects belonging, as alleged, to the Company, and for an account. The case was heard upon a bill, answer, and testimony, and there was a decree in favor of the Company in the Circuit Court for the District of Rhode Island, in September, 1870. Upon appeal to this court the decree below was affirmed on the 6th of May, 1872, but the opinion has not been read. The defense was conducted by counsel originally employed and paid by Newbrough, under whom Gardner was licensee. On the first of July, 1869, before the decree in the Circuit Court, Newbrough and the Company compromised all matters of difference between them, with the understanding that this suit should go on to the final hearing and determination both in the Circuit Court and in this court on appeal, as if the compromise had not been made.

*The Company, however, paid the counsel employed for the defense as well as for themselves in Circuit Court, and subsequently in this court. These facts appear from the record and from the admissions of the Company in the ninth article of their answer to the motion to dismiss the appeal. They are the only facts which we think it necessary to notice.*

It may be that the Company has not become the legal or equitable owners of the opposing interests involved in the suit. There may be, and doubtless are, large opposing interests of which they are neither the legal nor equitable owners. But it can not be admitted that one party to a suit can pay the fees of counsel on both sides, both in the court below and on appeal, without being held to have such control over both the preparation and argument of the cause as to make the suit merely collusive in both courts. It can make no difference that the counsel fees were charged to the party apparently, though not really liable to pay them, and payment from the other party procured him. This, indeed, is a circumstance against the party who pays the fees rather than in his favor.

The motion to vacate the decree of affirmance heretofore made, and to dismiss the appeal, must, therefore, be granted, and an order made to recall the mandate which has been issued to the Circuit Court. We take occasion, however, to say that we see nothing in the conduct of the counsel who actually represented the Company which merits blame, or which ought to effect in any degree the high esteem in which they have been held. Neither of them appears to have had any krowledge of any arrangements made by their client with the opposing party.

True copy.

[SEAL.] Test: D. W. MIDDLETON,
*Clerk Supreme Court U. S.*

---

[That there may be no question as to the scope and effect of the foregoing decision, we have procured and appended a copy of the mandate which *had been issued* to the Circuit Court, and which is *recalled* and *annulled* by the decree on the motion to dismiss the appeal.]

UNITED STATES OF AMERICA, ss.

THE PRESIDENT OF THE UNITED STATES OF AMERICA,

*To the Honorable the Judges of the Circuit Court of the United States for the District of Rhode Island, Greeting:*

*Whereas*, lately, in the Circuit Court of the United States for the District of Rhode Island, before you, or some of you, in a cause between the Goodyear Dental Vulcanite Company and Josiah Bacon, complainants, and Benoni E. Gardner, respondent, wherein the decree of the said Circuit Court entered in said cause on the 8th day of September, A. D. 1870, is in the following words, viz:

"Upon the coming in of the master's report, and on motion of the complainant's counsel, it is ordered and decreed by the Court that the said report be confirmed and established, and that the said defendant, Benoni E. Gardner, and his agents and servants, be perpetually enjoined from making, using, or vending to others to be used, any one or more dentures or plates for artificial teeth manufactured according to the process described in the reissue patent, in said bill of complaint mentioned.

"And the Court doth further order and decree that the said defendant do forthwith pay unto the complainants the sum of seventy-five dollars, being the amount of profit found by the said master's report to have been received by the said defendant, Gardner, from the manufacture and sale of plates for artificial teeth manufactured according to the process described in the said reissue patent, in violation of the exclusive right of the complainants.

"And the Court doth further order and decree that the said defendant, Gardner, do pay to the said complainants the cost of this suit, taxed at two hundred and forty-eight dollars and eighty-four cents ($248.84), entered as a decree of this Court, per order thereof, of this day, September8th A. D. 1870."

As by the inspection of the transcript of the record of the said Circuit Court, which was brought into the Supreme Court of the United States by virtue of an appeal, agreeably to the act of Congress, in such case made and provided, fully and at large appears.

*And Whereas*, in the present term of December, in the year of our Lord one thousand eight hundred and seventy-one, the said cause came on to be heard before the said Supreme Court, on the said transcript of the record, and was argued by counsel: On consideration whereof, it is now here ordered, adjudged, and decreed by this Court that the decree of the said Circuit Court in this cause be and the same is hereby affirmed, with costs and interest until paid, at the same rate per annum that similar decrees bear in the courts of the State of Rhode Island, and that the said complainants recover against the said respondent, Benoni E. Gardner, three hundred and ninety-nine dollars and nineteen cents for their costs herein expended, and have execution therefor. *6th May*, 1873.

You, therefore, are hereby commanded that such execution and proceedings be had in said cause, as according to right and justice. and the laws of the United States, ought to be had, the said appeal notwithstanding.

Witness the Honorable Salmon P. Chase, Chief Justice of said Supreme Court, the first Monday of December, in the year of our Lord one thousand eight hundred and seventy-one.

Cost of Complainants:

| | |
|---|---|
| Clerk . . . . | $379.16 |
| Attorney . . | 20.00 |
| | $399.19 |

Taxed by D. W. Middleton,
C. S. C. U. S.

---

SUPREME COURT U. S. DECEMBER TERM, 1871.

Costs of the Goodyear Dental Vulcanite Company *et al.*, in No 133, 1870:

$379.19; Fee book L; pa. 378; Test.: D. W. Middleton,
C. S. C. U. S.

1872, May 14. Received payment, per A Pollok. Esq.
D. W. Middleton,
C. S. C. U. S.

---

The Ninth Annual Meeting of the Illinois State Dental Society will be held at Rock Island Ill., beginning on Tuesday, the thirteenth day of May, 1872.

Discussions will be held conjointly with the Iowa State Dental Society, and a very profitable and largely attended meeting is anticipated.

All are cordially invited to attend.

CHARLES R. E. KOCK, Sec'y.

### One-Eighth Objective.

At a recent meeting of the Quickett Microscopical Club, of University College, Mr. Thomas Powell exhibited an one-eightieth objective, made by himself. At a public meeting, with the time for examination limited, no decisive tests could be made, but so far as could be seen it performed well. It defined the podura scale well, without color or aberation of light. It magnified about 4,000 diameters with the A eye-piece.

### The Rubber Committee.

The Committee appointed by the Ohio State Dental Society, to look to the interests of the profession of the State in matters at issue with the Dental Vulcanite Company, held a meeting in this city on the 15th inst. In view of the fact that Mr. S. S. White has consented to take the entire management of the defense as far as possible, the courts have decided to issue in a few days an appeal to the dentists of Ohio, asking them to contribute in aid of the expenses which will necessarily be incurred in any other further litigation with this Company. Mr. White having given his time, and expended about five thousand dollars to obtain a favorable verdict in the Gardner appeal case, thus giving the profession an opportunity to again assert their rights, it is but just to him that the dental profession respond promptly and liberally to this call for pecuniary aid.

It is the purpose of the court, we learn, to recommend the organization in every city and county of the State of the dentists, so that there can be concert of action, and no time lost in assuming the defensive.

